# A Case of Abscess in the Groin, Attended with Symptoms of Hernia, from Which Two Lumbrici Teretes and Afterwards Fæcal Matter Were Discharged, and the Patient Recovered

**Published:** 1845-07

**Authors:** Thomas Howell

**Affiliations:** Risborough


*A case of abscess in the groin, attended with symptoms of Hernia, from which two lumbrici teretes and afterwards fæcal matter were discharged, and the patient recovered.* By THOMAS HOWELL, Esq., of Risborough.

John Stevenson, aged 70, was attacked with symptoms of strangulated hernia. The author found a small hard tumour, about the size of a hen's egg, in the right inguinal region, lying over Poupart's ligament, about midway between the internal and external abdominal rings. At first he supposed it to be a hernia, but on careful examination could detect no impulse on coughing, nor detect a neck towards the internal ring, and the tumour was not in the precise situation of inguinal hernia, and he was led to conclude that it was an enlarged inguinal gland. The symptoms were relieved by calomel and opium and a dose of castor oil, which acted freely. After two or three days phlegmonous inflammation took place, and on fluctuation being perceived the part was opened and four ounces of pus discharged. On the poultice being removed next morning, two worms (*lumbrici teretes*) each from five to six inches long, were found in it. After this, feculent matter continued to escape from the wound for three weeks. He also passed per anum fæces, but not of so bright a yellow colour as that fiom the groin. The discharge from the wound gradually diminished, and in five weeks the author succeeded, by means of pressure, in healing this orifice. The patient stated that he never had any hernia.

Mr. Cæsar Hawkins would allude to one point in the paper, particularly as he was afraid it had been a source of error in diagnosis in some other cases. He referred to the fact of the medical attendants of the case detailed having come to the conclusion that the tumour was not a strangulated hernia, in consequence of the absence of all impulse in it on the patient coughing. This was really one reason for concluding that the hernia was strangulated. In nine out of ten cases of strangulated hernia no impulse would be communicated to the swelling on coughing.

Dr. Chowne mentioned a case which bore some analogy to the one under consideration. It occurred some years since, and was one of constipation, to relieve which the patient swallowed a quantity of quick-silver. A tumour subsequently formed in the groin, and opened by sloughing. Two ounces of quick-silver came away, with fæcal matter, from the opening. The symptoms presented were similar to those in the case under consideration. The patient eventually did well.

Mr. Arnott, some years since, had a case under his care at the Middlesex Hospital. The patient, a young girl, had an abscess formed on the right side, in the situation of that in Mr. Howell's case: the integuments sloughed, and there was a discharge of fæcal matter. The patient recovered. He agreed with Mr. Hawkins respecting the absence of impulse in the tumour on coughing, in strangulated hernia. The last case of hernia on which he had operated was an instance of this kind. The patient was an old woman, labouring under strangulated femoral hernia. Coughing imparted no impulse to the tumour. He pointed out this peculiarity to the students, and explained his reasons for operating. The patient recovered from the proceeding. The surgeon was often puzzled in reference to abscesses having communication with the intestine. He had a short time since been consulted in a case of hernia, in which an opening had been made into a tumour situated in the groin. and which had been decided not to be hernial, because there was no impulse imparted to it on coughing. The surgeon was surprised to find a quantity of fæcal matter follow the knife, and he became alarmed. The abscess was on the left, not, as usual, on the right side. No hernia existed, but there was, no doubt, a communication between the abscess and intestine. The patient recovered.

*Dub. Med. Press.*

